# PReS-FINAL-2304: Systemic lupus erythematosus in childhood: experience of single center (data of last ten years)

**DOI:** 10.1186/1546-0096-11-S2-P294

**Published:** 2013-12-05

**Authors:** O Kasapcopur, S Sahin, K Barut, N Canpolat, S Caliskan, L Sever, N Arisoy

**Affiliations:** 1 Pediatric Rheumatology, Istanbul University, Cerrahpasa Medical Faculty, Istanbul, Turkey; 2 Pediatric Nephrology, Istanbul University, Cerrahpasa Medical Faculty, Istanbul, Turkey

## Introduction

Systemic lupus erythematosus (SLE) is a multisystem disease that is rarely seen in childhood. Diagnosing this disease in childhood is a challenge since it has a variable presentation. The progress of the disease also varies during childhood.

## Objectives

We aimed to review the clinical and laboratory findings and disease progress of the patients who were diagnosed as juvenile onset SLE in our pediatric rheumatology department during the last 10 years.

## Methods

Ninety subjects who were diagnosed as juvenile onset SLE according to the 1982 ACR SLE classification criteria during the last 10 years were included in the study. All of the patients had a follow-up period of at least 1 year. The clinical findings, progress of the disease and response to treatment were all recorded retrospectively from patient files.

## Results

Of the 90 subjects, 79 were female and 11 were male. Mean age at the onset of disease was 10.2 ± 2.9 years (range 3.1-16 years), and mean age at the time of diagnosis was 11.6 ± 3 years (range 3,8-16 years). Twenty two (24.4%) of the subjects had been followed up initially with a different diagnosis. Eighty (88.8%) of the patients had malar rash, 32 (35.6%) had oral ulcer, 55 (61.1%) had a non-deforming polyarthritis, 12 (13.3%) had serositis (7 pericarditis and 5 pleuritis). None of the patients developed avascular necrosis. Thirty eight (42.2%) of the patients had renal involvement. Of the patients with renal involvement, 5 progressed to end stage renal disease requiring hemodialysis. These 5 patients were those who were noncompliant with their periodic controls. Seventeen (18.8%) patients had neurologic involvement. None of the patients developed any irreversible neurologic sequela. At the onset of diagnosis, 88 (97.7%) of the patients had ANA positivity. Twelve (13.3%) of the patients had a family history of autoimmune disease. Ten patients developed secondary antiphospholipid syndrome during follow up. All of the patients were given corticosteroid treatment at varying doses. Patients with renal involvement were given cyclophosphamide therapy. Azathioprine and mycophenolate mofetil were used in 62 and 19 patients, respectively. The mean durations of follow up were 62 months (range 12-120 months). Three patients died during the follow up period (due to sepsis, antiphospholipid syndrome, eosaphageal varices hemorrhage). Patients who were over 20 years of age were transferred to adult rheumatology department.

## Conclusion

Juvenile onset SLE is a disease that can present with different and varying findings. Complications can be lessened and long term sequela can be prevented with close and effective treatment and follow up of the disease.

## Disclosure of interest

None declared.

